# Infinite time interval backward stochastic differential equations with continuous coefficients

**DOI:** 10.1186/s40064-016-3419-3

**Published:** 2016-10-06

**Authors:** Zhaojun Zong, Feng Hu

**Affiliations:** School of Statistics, Qufu Normal University, Qufu, 273165 China

**Keywords:** Backward stochastic, Differential equation (BSDE), Linear growth condition, Comparison theorem, 60H10

## Abstract

In this paper, we study the existence theorem for $L^{p}$ $(1<p<2)$ solutions to a class of 1-dimensional infinite time interval backward stochastic differential equations (BSDEs) under the conditions that the coefficients are continuous and have linear growths. We also obtain the existence of a minimal solution. Furthermore, we study the existence and uniqueness theorem for $L^{p}$ $(1<p<2)$ solutions of infinite time interval BSDEs with non-uniformly Lipschitz coefficients. It should be pointed out that the assumptions of this result is weaker than that of Theorem 3.1 in Zong (Turkish J Math 37:704–718, 2013).

## Background

The theory of nonlinear backward stochastic differential equations (BSDEs for short) was developed by Pardoux and Peng (1990), from which we know that there exists a unique adapted and square integrable solution to a BSDE of the type

1 $$\begin{aligned} y_t=\xi +\int _t^Tg(s,y_s,z_s)\mathrm{d}s-\int _t^Tz_s\mathrm{d}W_s, \ \ \ \ t\in [0,T], \end{aligned}$$

provided the function *g* (also called the generator) is Lipschitz in both variables *y* and *z*, and $\xi$ and $(g(t,0,0))_{0\le t\le T}$ are square integrable. The theory of BSDEs is very useful, due to the connection of this subject with mathematical finance, stochastic control, partial differential equation, stochastic game and stochastic geometry and mathematical economics. Later, many researchers developed the theory of BSDEs and their applications in a series of papers (for example, see Briand et al. (2003), Lepeltier and San Martin (1997), Pardoux (1997, 1998), Karoui et al. (1997) and the references therein) under some other assumptions on the coefficients but for a fixed terminal time $T>0$. Let us mention the contribution of Lepeltier and San Martin (1997). In Lepeltier and San Martin (1997), the authors got the existence of a solution for a 1-dimensional BSDE where the coefficient was continuous, it had linear growth, and the terminal condition was square integrable. They also obtained the existence of a minimal solution.

Chen and Wang (2000) obtained the existence and uniqueness theorem for $L^2$ solutions of BSDEs with non-uniformly Lipschitz coefficients when $T\equiv \infty$, by the martingale representation theorem and fixed point theorem. In fact, such a problem has been investigated by Peng (1990), Pardoux (1997), Darling and Pardoux (1997) and other researchers under the assumption that terminal value $\xi =0$ or $E[\mathrm{e}^{p\rho T}|\xi |^p]<\infty$ for some constant $\rho$ and random terminal time *T* (i.e., *T* is a stopping time). But in $L^{p}$ ($1<p<2$), there is no the martingale representation theorem. Zong (2013) studied $L^{p}$ solutions to infinite time interval BSDEs with non-uniformly Lipschitz coefficients. She gave a new a priori estimate. By using this a priori estimate, she got the existence and uniqueness of $L^{p}$ solutions to infinite time interval BSDEs.

In this paper, we study the existence theorem for $L^{p}$ ($1<p<2$) solutions to a class of 1-dimensional infinite time interval BSDEs under the conditions that the coefficients are continuous and have linear growths. We also obtain the existence of a minimal solution. Furthermore, we study the existence and uniqueness theorem for $L^{p}$ $(1<p<2)$ solutions of infinite time interval BSDEs with non-uniformly Lipschitz coefficients. It should be pointed out that the assumptions of this result is weaker than that of Theorem 3.1 in Zong (2013).

This paper is organized as follows. In “Preliminaries” section, we introduce some notations, assumptions and lemmas. In “Main results and proofs” section, we give our main results including the proofs.

## Preliminaries

In this section, we shall present some notations, assumptions and lemmas that are used in this paper.

**Notation.** The Euclidean norm of a vector $x\in {R^{k}}$ will be denoted by |*x*|, and for a $k\times d$ matrix *A*, we define $||A||=\sqrt{TrAA^{*}}$, where $A^{*}$ is the transpose of *A*.

Let $(\Omega ,{\mathcal {F}},P)$ be a completed probability space, $(W_t)_{t\ge 0}$ be a *d*-dimensional standard Brownian motion defined on this space and $({\mathcal {F}}_t)_{t\ge 0}$ be the natural filtration generated by Brownian motion $(W_t)_{t\ge 0}$, that is

$$\begin{aligned} {{\mathcal {F}}_t}:=\sigma \{W_s;s\le t\}\vee {\mathcal {N}}, \end{aligned}$$

where ${\mathcal {N}}$ is the set of all *P*-null subsets. Furthermore, we define ${\mathcal {F}}:=\sigma \left( \bigcup \nolimits _{t\ge 0}{{\mathcal {F}}_t}\right)$.

We consider the following spaces:

$L^{p}(\Omega ,{\mathcal {F}},P,{R}^k):=\{\xi :\xi$ is ${R}^k$-valued and ${\mathcal {F}}$-measurable random variable such that $E[|\xi |^p]<\infty ,p\ge 1\}$;

$${\mathcal L}(\Omega ,{\mathcal {F}},P,{R}^k):=\bigcup \nolimits _{p>1}L^{p}(\Omega ,{\mathcal {F}},P,{R}^k)$$;

${\mathcal { S}}^p({R}^k):=\{V:V_t$ is ${R}^k$-valued and ${\mathcal {F}}_t$-adapted process such that $E[\sup _{t\ge 0}|V_t|^p]<\infty ,p\ge 1\}$;

$${\mathcal { S}}({R}^k):=\bigcup \nolimits _{p>1}{\mathcal { S}}^p({R}^k)$$;

${\mathcal { L}}^p({R}^{k\times d}):=\{V:V_t$ is ${R}^{k\times d}$-valued and ${\mathcal {F}}_t$-adapted process such that $E[(\int _0^\infty ||V_s||^2\mathrm{d}s)^\frac{p}{2}]<\infty , p\ge 1\}$;

$${\mathcal { L}}({R}^{k\times d}):=\bigcup \nolimits _{p>1}\mathcal{L}^p({R}^{k\times d})$$.

In the sequel, we assume that $1<p<2$.

Consider the following infinite time interval BSDE

2 $$\begin{aligned} Y_t=\xi +\int _t^\infty g(s,Y_s,Z_s)\mathrm{d}s-\int _t^\infty Z_s \mathrm{d}W_s. \end{aligned}$$

Let

$$\begin{aligned} g:\Omega \times {R}_{+}\times {R}^k\times {R}^{k\times d}\mapsto {R}^k \end{aligned}$$

such that for any $(y,z)\in {R}^k\times {R}^{k\times d}$, $g(\cdot ,y,z)$ is ${\mathcal {F}}_t$-progressively measurable. We make the following assumptions:

(A.1) There exist two positive non-random functions $\alpha (t)$ and $\beta (t)$, such that for all $y_1,y_2\in {R}^k$, $z_1,z_2\in {R}^{k\times d}$,

$$\begin{aligned} \left| g(t,y_1,z_1)-g(t,y_2,z_2)\right| \le \alpha (t)\left| y_1-y_2\right| +\beta (t)||z_1-z_2||, \end{aligned}$$

where $\alpha (t)$ and $\beta (t)$ satisfy that $\int _0^\infty \alpha (t)\mathrm{d}t<\infty$, $\int _0^\infty \beta ^2(t)\mathrm{d}t<\infty$;

(A.$1^{'}$) There exist two positive non-random functions $\alpha (t)$ and $\beta (t)$, such that for all $y_1,y_2\in {R}^k$, $z_1,z_2\in {R}^{k\times d}$,

$$\begin{aligned}\left| g(t,y_1,z_1)-g(t,y_2,z_2)\right| \le \alpha (t)\left| y_1-y_2\right| +\beta (t)||z_1-z_2||,\end{aligned}$$

where $\alpha (t)$ and $\beta (t)$ satisfy that $\int _0^\infty \alpha (t)\mathrm{d}t<\infty$, $\int _0^\infty \beta (t)\mathrm{d}t<\infty$, $\int _0^\infty \beta ^2(t)\mathrm{d}t<\infty$;

(A.2) $$E\left[ \left( \int _0^\infty |g(t,0,0)|\mathrm{d}t\right) ^p\right] <\infty$$;

(A.$2^{'}$) There exists some constant $T\in [0,\infty )$ such that

$$\begin{aligned}&E\left[ \left( \int _0^T|g(t,0,0)|\mathrm{d}t\right) ^p\right]<\infty ,\\&E\left[ \left( \int _T^\infty |g(t,0,0)|\mathrm{d}t\right) ^2\right] <\infty ; \end{aligned}$$

(A.3) Linear growth: There exists a positive non-random function $\gamma (t)$ such that

$$\begin{aligned} |g(\omega ,t,y,z)|\le \gamma (t)(1+|y|+||z||),\ \ \forall (\omega ,t,y,z) \in \Omega \times {R}_{+}\times {R}^k\times {R}^{k\times d} \end{aligned}$$

where $\gamma (t)$ satisfies that $\int _0^\infty \gamma (t)\mathrm{d}t<\infty$, $\int _0^\infty \gamma ^2(t)\mathrm{d}t<\infty$;

(A.4) For fixed $\omega$ and *t*, $g(\omega ,t,\cdot ,\cdot )$ is continuous.

### **Lemma 1**

(see Zong 2013) *Under assumptions* (A.$1^{'}$) *and *(A.$2^{'}$), *if* $\xi \in L^{p}(\Omega ,{\mathcal {F}},P,{R}^k)$, *then BSDE* (2) *has a unique solution* $(Y,Z)\in \mathcal{S}^p({R}^k)\times {\mathcal { L}}^p({R}^{k\times d})$.

## Main results and proofs

In this section, first we study the existence and uniqueness theorem for $L^{p}$ solutions of infinite time interval BSDEs with non-uniformly Lipschitz coefficients. It should be pointed out that the assumptions of this result is weaker than that of Lemma  1.

### **Theorem 2**

*Under assumptions (A.1) and (A.2), if* $\xi \in L^{p}(\Omega ,{\mathcal {F}},P,{R}^k)$, *then BSDE* (2) *has a unique solution* $(Y,Z)\in {\mathcal { S}}^p({R}^k)\times {\mathcal { L}}^p({R}^{k\times d})$.

In order to prove Theorem  2, we give an a priori estimate.

### **Lemma 3**

*Suppose that (A.1) holds for* *g*. *Furthermore, each* $\phi _i$ ($i=1,2$) *satisfies that*

$$\begin{aligned} E\left[ \left( \int _0^\infty |\phi _i(s)|\mathrm{d}s\right) ^p\right] <\infty . \end{aligned}$$

*For any* $T\in [0,\infty ]$, *let* $\xi _i\in L^{p}(\Omega ,{\mathcal {F}}_T,P,{R}^k)$, $(Y^i,Z^i)\in {\mathcal { S}}^p({R}^k)\times \mathcal{L}^p({R}^{k\times d})$ *satisfy the following BSDEs:*

$$\begin{aligned} Y_t^i=\xi _i+\int _t^T \left[ g\left( s,Y_s^i,Z_s^i\right) +\phi _i(s)\right] \mathrm{d}s -\int _t^T Z_s^i \mathrm{d}W_s,\ \ \ i=1,2. \end{aligned}$$

*Then there exists a positive constant* $C_p$ *depending only on* *p* *such that, for any* $\tau \in [0,T]$,

3 $$\begin{aligned}&E\left[ \sup \limits _{s\in [\tau ,T]}\left| Y_s^1-Y_s^2\right| ^p +\left( \int _\tau ^T\left| \left| Z_s^1-Z_s^2\right| \right| ^2\mathrm{d}s\right) ^\frac{p}{2}\right] \nonumber \\&\quad \le C_pE\left[ \left| \xi _1-\xi _2\right| ^p+\left( \int _0^\infty |\phi _1(s)-\phi _2(s)|\mathrm{d}s\right) ^p\right] \nonumber \\&\qquad + C_pl_{(\tau ,T]}E\left[ \sup \limits _{s\in [\tau ,T]}\left| Y_s^1-Y_s^2\right| ^p +\left( \int _\tau ^T\left| \left| Z_s^1-Z_s^2\right| \right| ^2\mathrm{d}s\right) ^\frac{p}{2}\right] , \end{aligned}$$

*where* $$l_{(\tau ,T]}= \left( \int _\tau ^T\alpha (s)\mathrm{d}s+\int _\tau ^T\beta ^2(s)\mathrm{d}s\right) ^{\frac{p}{2}} +\left( \int _\tau ^T\alpha (s)\mathrm{d}s\right) ^p$$.

### *Proof*

Applying Itô’s formula to $\left| Y_t^1-Y_t^2\right| ^2$, we have

4 $$\begin{aligned}&\left| Y_\tau ^1-Y_\tau ^2\right| ^2+\int _\tau ^T\left| \left| Z_s^1-Z_s^2\right| \right| ^2\mathrm{d}s\nonumber \\&\quad =|\xi _1-\xi _2|^2+2\int _\tau ^T\left<Y_s^1-Y_s^2, \left( g\left( s,Y_s^1,Z_s^1\right) -g\left( s,Y_s^2,Z_s^2\right) +\phi _1(s)-\phi _2(s)\right) \right> \mathrm{d}s\nonumber \\&\qquad -2\int _\tau ^T\left<Y_s^1-Y_s^2,\left( Z_s^1-Z_s^2\right) \mathrm{d}W_s\right>. \end{aligned}$$

From the Lipschitz assumption (A.1) on *g*, we have

5 $$\begin{aligned}&2\left<Y_s^1-Y_s^2,\left( g\left( s,Y_s^1,Z_s^1\right) -g\left( s,Y_s^2,Z_s^2\right) \right) \right>\nonumber \\&\quad \le 2\alpha (s)\left| Y_s^1-Y_s^2\right| ^2+2\beta (s)\left| Y_s^1-Y_s^2\right| \left| \left| Z_s^1-Z_s^2\right| \right| \nonumber \\&\quad \le 2\alpha (s)\left| Y_s^1-Y_s^2\right| ^2+2\beta ^2(s)\left| Y_s^1-Y_s^2\right| ^2+\frac{1}{2}\left| \left| Z_s^1-Z_s^2\right| \right| ^2\nonumber \\&\quad \le 2\left( \alpha (s)+\beta ^2(s)\right) \sup \limits _{s\in [\tau ,T]}\left| Y_s^1-Y_s^2\right| ^2 +\frac{1}{2}\left| \left| Z_s^1-Z_s^2\right| \right| ^2. \end{aligned}$$

It follows that

6 $$\begin{aligned}&\frac{1}{2}\int _\tau ^T\left| \left| Z_s^1-Z_s^2\right| \right| ^2\mathrm{d}s\nonumber \\&\quad \le |\xi _1-\xi _2|^2+2\left( \int _\tau ^T\alpha (s)\mathrm{d}s+\int _\tau ^T\beta ^2(s)\mathrm{d}s\right) \sup \limits _{s\in [\tau ,T]}\left| Y_s^1-Y_s^2\right| ^2\nonumber \\&\qquad +2\int _\tau ^T\left| Y_s^1-Y_s^2\right| |\phi _1(s)-\phi _2(s)| \mathrm{d}s+2\left| \int _\tau ^T\left<Y_s^1-Y_s^2,\left( Z_s^1-Z_s^2\right) \mathrm{d}W_s\right>\right| . \end{aligned}$$

Since $2\int _\tau ^T\left| Y_s^1-Y_s^2\right| |\phi _1(s)-\phi _2(s)|\mathrm{d}s\le \sup \limits _{s\in [\tau ,T]}\left| Y_s^1-Y_s^2\right| ^2+\left( \int _0^\infty |\phi _1(s)-\phi _2(s)|\mathrm{d}s\right) ^2$, we have

7 $$\begin{aligned}&\int _\tau ^T\left| \left| Z_s^1-Z_s^2\right| \right| ^2\mathrm{d}s\nonumber \\&\quad \le 4\left( |\xi _1-\xi _2|^2+\left( \int _0^\infty |\phi _1(s)-\phi _2(s)|\mathrm{d}s\right) ^2\right) \nonumber \\&\qquad +4\left( 1+\int _\tau ^T\alpha (s)\mathrm{d}s+\int _\tau ^T\beta ^2(s)\mathrm{d}s\right) \sup \limits _{s\in [\tau ,T]}\left| Y_s^1-Y_s^2\right| ^2\nonumber \\&\qquad +4\left| \int _\tau ^T\left<Y_s^1-Y_s^2,\left( Z_s^1-Z_s^2\right) \mathrm{d}W_s\right>\right| . \end{aligned}$$

Using the following fact: if *b*, $a_i\ge 0$ and $b\le \sum \nolimits _{i=1}^na_i$, then $b^p\le \sum \nolimits _{i=1}^na_i^p$ for any $p\in (0,1)$, we have

8 $$\begin{aligned}&\left( \int _\tau ^T\left| \left| Z_s^1-Z_s^2\right| \right| ^2\mathrm{d}s\right) ^{\frac{p}{2}}\nonumber \\&\quad \le c_p\left( |\xi _1-\xi _2|^p+\left( \int _0^\infty |\phi _1(s)-\phi _2(s)|\mathrm{d}s\right) ^p\right) \nonumber \\&\qquad + c_p\left[ 1+\left( \int _\tau ^T\alpha (s)\mathrm{d}s+\int _\tau ^T\beta ^2(s)\mathrm{d}s\right) ^{\frac{p}{2}}\right] \sup \limits _{s\in [\tau ,T]}\left| Y_s^1-Y_s^2\right| ^p\nonumber \\&\qquad + c_p\left( \left| \int _\tau ^T(Y_s^1-Y_s^2)(Z_s^1-Z_s^2)\mathrm{d}W_s\right| ^{\frac{p}{2}}\right) , \end{aligned}$$

where $c_p$ is a positive constant depending only on *p*. By the Burkholder–Davis–Gundy inequality, we get

9 $$\begin{aligned}&c_pE\left[ \left| \int _\tau ^T\left<Y_s^1-Y_s^2,\left( Z_s^1-Z_s^2\right) \mathrm{d}W_s\right>\right| ^\frac{p}{2}\right] \nonumber \\&\quad \le d_pE\left[ \left( \int _\tau ^T\left| Y_s^1-Y_s^2\right| ^2\left| \left| Z_s^1-Z_s^2\right| \right| ^2\mathrm{d}s\right) ^\frac{p}{4}\right] \nonumber \\&\quad \le d_pE\left[ \sup \limits _{s\in [\tau ,T]}\left| Y_s^1-Y_s^2\right| ^{\frac{p}{2}}\left( \int _\tau ^T\left| \left| Z_s^1-Z_s^2\right| \right| ^2\mathrm{d}s\right) ^\frac{p}{4}\right] \end{aligned}$$

and thus

10 $$\begin{aligned} c_pE\left[ \left| \int _\tau ^T\left<Y_s^1-Y_s^2,\left( Z_s^1-Z_s^2\right) \mathrm{d}W_s\right>\right| ^\frac{p}{2}\right]\le \,& \frac{1}{2}E\left[ \left( \int _\tau ^T\left| \left| Z_s^1-Z_s^2\right| \right| ^2\mathrm{d}s\right) ^\frac{p}{2}\right] \nonumber \\&+\frac{d_p^2}{2}E\left[ \sup \limits _{s\in [\tau ,T]}\left| Y_s^1-Y_s^2\right| ^p\right] , \end{aligned}$$

where $d_p$ is a positive constant depending only on *p*. From (8) and (10), we have

11 $$\begin{aligned}&E\left[ \left( \int _\tau ^T\left| \left| Z_s^1-Z_s^2\right| \right| ^2\mathrm{d}s\right) ^\frac{p}{2}\right] \nonumber \\&\quad \le C\left( E[|\xi _1-\xi _2|^p]+E\left[ \left( \int _0^\infty |\phi _1(s)-\phi _2(s)|\mathrm{d}s\right) ^p\right] \right) \nonumber \\&\qquad + C\left( 1+l_{(\tau ,T]}\right) E\left[ \sup \limits _{s\in [\tau ,T]}\left| Y_s^1-Y_s^2\right| ^p \right] , \end{aligned}$$

where *C* is a positive constant depending only on *p*.

On the other hand, we prove

12 $$\begin{aligned}&E\left[ \sup \limits _{s\in [\tau ,T]}\left| Y_s^1-Y_s^2\right| ^p \right] \nonumber \\&\quad \le C^{'}E\left[ \left| \xi _1-\xi _2\right| ^p+\left( \int _0^\infty |\phi _1(s)-\phi _2(s)|\mathrm{d}s\right) ^p\right] \nonumber \\&\qquad + C^{'}l_{(\tau ,T]}E\left[ \sup \limits _{s\in [\tau ,T]}\left| Y_s^1-Y_s^2\right| ^p +\left( \int _\tau ^T\left| \left| Z_s^1-Z_s^2\right| \right| ^2\mathrm{d}s\right) ^\frac{p}{2}\right] , \end{aligned}$$

where $C^{'}$ is a positive constant depending only on *p*. Obviously, $\left\{ \int _\tau ^t\left( Z_s^1-Z_s^2\right) \mathrm{d}W_s;\tau \le t\le T\right\}$ is an ${\mathcal {F}}_t$-martingale. Thus, it follows that

13 $$\begin{aligned}&Y_t^1-Y_t^2 =E\left[ (\xi _1-\xi _2)+\int _t^T\left( g\left( s,Y_s^1,Z_s^1\right) -g\left( s,Y_s^2,Z_s^2\right) +\phi _1(s)-\phi _2(s)\right) \mathrm{d}s|{\mathcal {F}}_t\right] . \end{aligned}$$

Applying Doob’s inequality, we can deduce that

14 $$\begin{aligned}&E\left[ \sup \limits _{t\in [\tau ,T]}\left| Y_t^1-Y_t^2\right| ^p \right] \nonumber \\&\quad \le E\left[ \sup \limits _{t\in [\tau ,T]}\left( E\left[ |\xi _1-\xi _2| +\int _\tau ^T\left| g\left( s,Y_s^1,Z_s^1\right) -g\left( s,Y_s^2,Z_s^2\right) +\phi _1(s)-\phi _2(s)\right| \mathrm{d}s|{\mathcal {F}}_t\right] \right) ^p\right] \nonumber \\&\quad \le \left( \frac{p}{p-1}\right) ^pE\left[ \left( |\xi _1-\xi _2| +\int _\tau ^T\left| g\left( s,Y_s^1,Z_s^1\right) -g\left( s,Y_s^2,Z_s^2\right) +\phi _1(s)-\phi _2(s)\right| \mathrm{d}s\right) ^p\right] \nonumber \\&\quad \le D_pE\left[ |\xi _1-\xi _2|^p+\left( \int _0^\infty \left| \phi _1(s)-\phi _2(s)\right| \mathrm{d}s\right) ^p+\left( \int _\tau ^T\left| g\left( s,Y_s^1,Z_s^1\right) -g\left( s,Y_s^2,Z_s^2\right) \right| \mathrm{d}s\right) ^p\right] , \end{aligned}$$

where $D_p$ is a positive constant depending only on *p*. From the Lipschitz assumption (A.1) on *g*, we have

15 $$\begin{aligned}&E\left[ \left( \int _\tau ^T\left| g\left( s,Y_s^1,Z_s^1\right) -g\left( s,Y_s^2,Z_s^2\right) \right| \mathrm{d}s\right) ^p\right] \nonumber \\&\quad \le E\left[ \left( \int _\tau ^T\left( \alpha (s)\left| Y_s^1-Y_s^2\right| +\beta (s)\left| \left| Z_s^1-Z_s^1\right| \right| \right) \mathrm{d}s\right) ^p\right] \nonumber \\&\quad \le M_p\left( \int _\tau ^T\alpha (s)\mathrm{d}s\right) ^pE\left[ \sup \limits _{s\in [\tau ,T]}\left| Y_s^1-Y_s^2\right| ^p \right] \nonumber \\&\qquad +M_p\left( \int _\tau ^T\beta ^2(s)\mathrm{d}s\right) ^{\frac{p}{2}}E\left[ \left( \int _\tau ^T\left| \left| Z_s^1-Z_s^2\right| \right| ^2\mathrm{d}s\right) ^\frac{p}{2}\right] , \end{aligned}$$

where $M_p$ is a positive constant depending only on *p*. From (14) and (15), we have

16 $$\begin{aligned}&E\left[ \sup \limits _{s\in [\tau ,T]}\left| Y_s^1-Y_s^2\right| ^p \right] \nonumber \\&\quad \le C^{'}E\left[ \left| \xi _1-\xi _2\right| ^p+\left( \int _0^\infty |\phi _1(s)-\phi _2(s)|\mathrm{d}s\right) ^p\right] \nonumber \\&\qquad + C^{'}l_{(\tau ,T]}E\left[ \sup \limits _{s\in [\tau ,T]}\left| Y_s^1-Y_s^2\right| ^p +\left( \int _\tau ^T\left| \left| Z_s^1-Z_s^2\right| \right| ^2\mathrm{d}s\right) ^\frac{p}{2}\right] , \end{aligned}$$

where $C^{'}$ is a positive constant depending only on *p*.

Combining (11) with (16), we get

17 $$\begin{aligned}&E\left[ \sup \limits _{s\in [\tau ,T]}\left| Y_s^1-Y_s^2\right| ^p +\left( \int _\tau ^T\left| Z_s^1-Z_s^2\right| ^2\mathrm{d}s\right) ^\frac{p}{2}\right] \nonumber \\&\quad \le C_pE\left[ \left| \xi _1-\xi _2\right| ^p+\left( \int _0^\infty |\phi _1(s)-\phi _2(s)|\mathrm{d}s\right) ^p\right] \nonumber \\&\qquad + C_pl_{(\tau ,T]}E\left[ \sup \limits _{s\in [\tau ,T]}\left| Y_s^1-Y_s^2\right| ^p +\left( \int _\tau ^T\left| \left| Z_s^1-Z_s^2\right| \right| ^2\mathrm{d}s\right) ^\frac{p}{2}\right] , \end{aligned}$$

where $C_p$ is a positive constant depending only on *p*. The proof of Lemma  3 is complete. $\square$

### Proof of Theorem  2

Let $\xi ^n:=(\xi \wedge n)\vee (-n)$ and $g_n(t,y,z):=g(t,y,z)-g(t,0,0)+h_n(g(t,0,0))$, where $h_n(g(t,0,0)):=\frac{g(t,0,0)n\mathrm{e^{-t}}}{|g(t,0,0)|\vee \left( n\mathrm{e^{-t}}\right) }$. By Theorem 1.2 in Chen and Wang (2000), BSDE

$$\begin{aligned} Y_t^n=\xi ^n+\int _t^\infty g_n\left( s,Y_s^n,Z_s^n\right) \mathrm{d}s-\int _t^\infty Z_s^n\mathrm{d}W_s \end{aligned}$$

has a unique solution $(Y^n,Z^n)\in {\mathcal { S}}^2({R}^k)\times \mathcal{L}^2({R}^{k\times d})$. Since

$$\begin{aligned} \left( \int _0^\infty \alpha (s)\mathrm{d}s+\int _0^\infty \beta ^2(s)\mathrm{d}s\right) ^{\frac{p}{2}} +\left( \int _0^\infty \alpha (s)\mathrm{d}s\right) ^p<\infty , \end{aligned}$$

we can choose a strictly increasing sequence $0=t_0<t_1<\cdots<t_{N}<t_{N+1}=\infty$, such that

$$\begin{aligned} l_{(t_i,t_{i+1}]}\le \frac{1}{2C_p}, \quad i=0,1,2,\ldots ,N. \end{aligned}$$

Applying Lemma  3, we have

18 $$\begin{aligned}&E\left[ \sup \limits _{s\in [t_i,t_{i+1}]}\left| Y_s^{m+n}-Y_s^n\right| ^p +\left( \int _{t_i}^{t_{i+1}}\left| \left| Z_s^{m+n}-Z_s^n\right| \right| ^2\mathrm{d}s\right) ^\frac{p}{2}\right] \nonumber \\&\quad \le C_pE\left[ \left| Y_{t_{i+1}}^{m+n}-Y_{t_{i+1}}^n\right| ^p\right] \nonumber \\&\qquad + C_pE\left[ \left( \int _0^\infty |h_{n+m}(g(s,0,0))-h_{n}(g(s,0,0))|\mathrm{d}s\right) ^p\right] \nonumber \\&\qquad +\frac{1}{2}E\left[ \sup \limits _{s\in [t_i,t_{i+1}]}\left| Y_s^{m+n}-Y_s^n\right| ^p +\left( \int _{t_i}^{t_{i+1}}\left| \left| Z_s^{m+n}-Z_s^n\right| \right| ^2\mathrm{d}s\right) ^\frac{p}{2}\right] . \end{aligned}$$

Thus

19 $$\begin{aligned}&E\left[ \sup \limits _{s\in [t_i,t_{i+1}]}\left| Y_s^{m+n}-Y_s^n\right| ^p +\left( \int _{t_i}^{t_{i+1}}\left| \left| Z_s^{m+n}-Z_s^n\right| \right| ^2\mathrm{d}s\right) ^\frac{p}{2}\right] \nonumber \\&\quad \le 2C_pE\left[ \left| Y_{t_{i+1}}^{m+n}-Y_{t_{i+1}}^n\right| ^p\right] \nonumber \\&\qquad + 2C_pE\left[ \left( \int _0^\infty |h_{n+m}(g(s,0,0))-h_{n}(g(s,0,0))|\mathrm{d}s\right) ^p\right] \nonumber \\&\quad \le 2C_pE\left[ \sup \limits _{s\in [t_{i+1},t_{i+2}]}\left| Y_s^{m+n}-Y_s^n\right| ^p +\left( \int _{t_{i+1}}^{t_{i+2}}\left| \left| Z_s^{m+n}-Z_s^n\right| \right| ^2\mathrm{d}s\right) ^\frac{p}{2}\right] \nonumber \\&\qquad + 2C_pE\left[ \left( \int _0^\infty |h_{n+m}(g(s,0,0))-h_{n}(g(s,0,0))|\mathrm{d}s\right) ^p\right], \quad i=0,1,2,\ldots ,N-1. \end{aligned}$$

In particulary, we have

20 $$\begin{aligned}&E\left[ \sup \limits _{s\ge t_N}\left| Y_s^{m+n}-Y_s^n\right| ^p +\left( \int _{t_N}^\infty \left| \left| Z_s^{m+n}-Z_s^n\right| \right| ^2\mathrm{d}s\right) ^\frac{p}{2}\right] \nonumber \\&\quad \le 2C_pE\left[ \left| \xi ^{m+n}-\xi ^n\right| ^p\right] \nonumber \\&\qquad + 2C_pE\left[ \left( \int _0^\infty |h_{n+m}(g(s,0,0))-h_{n}(g(s,0,0))|\mathrm{d}s\right) ^p\right] . \end{aligned}$$

From (19) and (20), it follows that

21 $$\begin{aligned} &E\left[ \sup \limits_{s\ge 0}\left| Y_s^{n+m}-Y_s^n\right|^p +\left( \int _0^\infty \left| \left| Z_s^{n+m}-Z_s^n\right| \right| ^2\mathrm{d}s\right)^\frac{p}{2}\right] \nonumber \\ &\quad \le \sum \limits _{i=0}^NE\left[ \sup \limits _{s\in [t_i,t_{i+1}]}\left| Y_s^{m+n}-Y_s^n\right| ^p +\left( \int\limits_{t_i}^{t_{i+1}}\left| \left| Z_s^{m+n}-Z_s^n\right| \right| ^2\mathrm{d}s\right) ^\frac{p}{2}\right] \nonumber \\ &\quad \le (2C_p+(2C_p)^2+\cdots +(2C_p)^{N+1})E\left[ \left| \xi ^{m+n}-\xi ^n\right| ^p\right] \nonumber \\ &\qquad + (N+1)(2C_p+(2C_p)^2+\cdots +(2C_p)^{N+1})E\left[ \left( \int\limits_0^\infty |h_{n+m}(g(s,0,0))-h_{n}(g(s,0,0))|\mathrm{d}s\right) ^p\right] \nonumber \\ &\quad \le \overline{C}E\left[ \left| \xi ^{m+n}-\xi ^n\right| ^p\right] \nonumber \\ &\qquad + \overline{C}E\left[ \left( \int\limits_0^\infty |h_{n+m}(g(s,0,0))-h_{n}(g(s,0,0))|\mathrm{d}s\right) ^p\right] , \end{aligned}$$

where $\overline{C}=(N+1)(2C_p+(2C_p)^2+\cdots +(2C_p)^{N+1})$. The right-hand side of Inequality (21) clearly tends to 0, as $n\rightarrow \infty$, uniformly in *m*, so we have a Cauchy sequence and the limit is a solution to BSDE (2). Let us consider (*Y*, *Z*) and $(Y^{'},Z^{'})$ to be two solutions to BSDE (2). In a similar manner to the proof of Inequality (21), we can obtain

$$\begin{aligned} E\left[ \sup \limits _{s\ge 0}\left| Y_s-Y_s^{'}\right| ^p+\left( \int _0^\infty \left| \left| Z_s-Z_s^{'}\right| \right| ^2\mathrm{d}s\right) ^\frac{p}{2}\right] \le 0. \end{aligned}$$

Thus, we get immediately $(Y,Z)=(Y^{'},Z^{'})$. The proof of Theorem  2 is complete. $\square$

### **Theorem 4**

(Comparison Theorem) *Assume that* $k=1$. *We make the same assumptions on* $\xi$, *g* *and* $\overline{\xi }$, $\overline{g}$ *as in Theorem*  2. *Let* $\left( \overline{Y},\overline{Z}\right)$ *be a solution of BSDE*

$$\begin{aligned} \overline{Y}_t=\overline{\xi }+\int _t^\infty \overline{g}\left( s,\overline{Y}_s,\overline{Z}_s\right) \mathrm{d}s-\int _t^\infty \overline{Z}_s\mathrm{d}W_s. \end{aligned}$$

*If we suppose that:*

22 $$\begin{aligned} \hat{\xi }:=\xi -\overline{\xi }\le 0,\ \ \ \hat{g}_t:=g\left( t,\overline{Y}_t,\overline{Z}_t\right) -\overline{g}\left( t,\overline{Y}_t,\overline{Z}_t\right) \le 0,\ \ \hbox {a.s.,} \end{aligned}$$

*then*

$$\begin{aligned} \hat{Y}_t:=Y_t-\overline{Y}_t\le 0,\ \ \hbox {a.s.,}\ \ \forall t\in [0,\infty ). \end{aligned}$$

*Moreover,* $\overline{Y}_t=Y_t$ *a.s., if and only if* $\overline{\xi }=\xi$ *a.s.,* $\overline{g}\left( t,Y_t,Z_t\right) =g\left( t,Y_t,Z_t\right)$ *a.s..*

### Proof

Suppose that $W_t=(W_t^1,W_t^2,\ldots ,W_t^d)^{T}$, $\forall t\in [0,\infty )$, where $W_t^i$ is the *i*th components of $W_t$. Let us consider the following BSDEs

$$\begin{aligned} Y_t & = \xi +\int _t^\infty g(s,Y_s,Z_s)\mathrm{d}s-\int _t^\infty Z_s \mathrm{d}W_s,\\ \overline{Y}_t &= \overline{\xi }+\int _t^\infty \overline{g}\left( s,\overline{Y}_s,\overline{Z}_s\right) \mathrm{d}s-\int _t^\infty \overline{Z}_s\mathrm{d}W_s, \end{aligned}$$

where $Z_t=(Z_t^1,Z_t^2,\ldots ,Z_t^d)^{T}$, $\overline{Z}_t=(\overline{Z}_t^1,\overline{Z}_t^2,\ldots ,\overline{Z}_t^d)^{T}$, $\forall t\in [0,\infty )$ and $\int _t^\infty Z_s \mathrm{d}W_s=\sum \nolimits _{i=1}^d\int _t^\infty Z_t^i\mathrm{d}W_t^i$, $\int _t^\infty \overline{Z}_s \mathrm{d}W_s=\sum \nolimits _{i=1}^d\int \limits _t^\infty \overline{Z}_t^i\mathrm{d}W_t^i$. Then, we have

23 $$\begin{aligned} \hat{Y}_t=\hat{\xi }+\int _t^\infty \left( a_s\hat{Y}_s+\left<b_s,\hat{Z}_s\right> +\hat{g}_s\right) \mathrm{d}s-\int _t^\infty \hat{Z}_s\mathrm{d}W_s, \end{aligned}$$

where

$$\begin{aligned} \hat{Z}_s=\, & Z_s-\overline{Z}_s =(Z_t^1-\overline{Z}_t^1,Z_t^2-\overline{Z}_t^2,\ldots ,Z_t^d-\overline{Z}_t^d)^T,\\ Z_s^{(i)}=\, & (\overline{Z}_s^1,\ldots ,\overline{Z}_s^i,Z_s^{i+1},\ldots ,Z_s^d)^T, \quad i=1,2,\ldots ,d-1,\\ Z_s^{(0)}=\, & Z_s=(Z_s^1,Z_s^2,\ldots ,Z_s^d)^{T},\\ Z_s^{(d)}=\, & \overline{Z}_s=(\overline{Z}_s^1,\overline{Z}_s^2,\ldots ,\overline{Z}_s^d)^{T},\\ a_s=\, & \frac{g\left( s,Y_s,Z_s\right) -g\left( s,\overline{Y}_s,Z_s\right) }{\hat{Y}_s}1_{\left\{ \hat{Y}_s\ne 0\right\} },\\ b_s^i=\, & \frac{g\left( s,\overline{Y}_s,Z_s^{(i-1)}\right) -g\left( s,\overline{Y}_s,Z_s^{(i)}\right) }{Z_s^i-\overline{Z}_s^i}1_{\left\{ Z_s^i-\overline{Z}_s^i\ne 0\right\} }, \quad i=1,2,\ldots ,d,\\ b_s=\, & (b_s^1,b_s^2,\ldots ,b_s^d)^T, \end{aligned}$$

which imply $$|a_s|\le \alpha (s)$$, $$|b_s|\le \beta (s)$$.

Solving (23), we know that the unique solution of BSDEs (23) can be represented as

24 $$\begin{aligned} \hat{Y}_t=E\left[ \hat{\xi }X_\infty +\int _t^\infty \hat{g}_sX_s\mathrm{d}s|{\mathcal {F}}_t\right] , \end{aligned}$$

where

$$\begin{aligned} X_s=\mathrm{exp}\left[ \int _t^s\left( a_r-\frac{1}{2}|b_r|^2\right) \mathrm{d}r+\int _t^sb_r\mathrm{d}W_r\right] ,\ \ \ s\ge t. \end{aligned}$$

From (24), we can obtain $\hat{Y}_t\le 0$, a.s. and if $\overline{\xi }=\xi$ a.s., $\overline{g}\left( t,Y_t,Z_t\right) =g\left( t,Y_t,Z_t\right)$ a.s., then $\overline{Y}_t=Y_t$ a.s..

Choosing $t=0$ in (24) and from the strict monotonicity of $E[\cdot ]$, we can obtain that if $\overline{Y}_0=Y_0$, then $\overline{\xi }=\xi$ a.s., $\overline{g}\left( t,Y_t,Z_t\right) =g\left( t,Y_t,Z_t\right)$ a.s.. The proof of Theorem  4 is complete. $\square$

Now we prove the existence theorem for $L^{p}$ solutions of 1-dimensional infinite time interval BDSDEs which generalizes Theorem 1 in Lepeltier and San Martin (1997).

### **Theorem 5**

*Assume that* $k=1$. *Under assumptions (A.3) and (A.4), if* $\xi \in L^{p}(\Omega ,{\mathcal {F}},P,{R})$, *then BSDE* (2) *has a solution* $(Y,Z)\in {\mathcal { S}}^p({R})\times {\mathcal { L}}^p({R}^d)$. *Also, there is a minimal solution* $(\underline{Y},\underline{Z})$ *of BSDE* (2), *in the sense that for any other solution* (*Y*, *Z*) *of *(2), *we have* $\underline{Y}\le Y$.

In order to prove Theorem  5, we need the following lemmas.

### **Lemma 6**

*Suppose that (A.3) and (A.4) hold for* *g*. *For each* $(\omega ,t,y,z)\in \Omega \times {R}_{+}\times {R}\times {R}^d$, *define the sequence of functions*

$$\begin{aligned} g_n(\omega ,t,y,z):=\inf \limits _{\left( y^{'},z^{'}\right) \in {Q}} \left\{ g(\omega ,t,y^{'},z^{'})+n\gamma (t)\left( \left| y-y^{'}\right| +\left| z-z^{'}\right| \right) \right\} , \end{aligned}$$

*where* *Q* *is the set of all rational numbers in* ${R}^{d+1}$. *Then* $g_n$ *satisfies*

(i) * Linear growth:* $$\forall (\omega ,t,y,z)\in \Omega \times {R}_{+}\times {R}\times {R}^d$$, $$|g_n(\omega ,t,y,z)|\le \gamma (t)(1+|y|+|z|)$$;
(ii) * Monotonicity in* *n*: $\forall (\omega ,t,y,z)\in \Omega \times {R}_{+}\times {R}\times {R}^d$, $g_n(\omega ,t,y,z)\uparrow$;
(iii) *Lipschitz condition:* $\forall (\omega ,t,y,z)$, $(\omega ,t,y^{'},z^{'})\in \Omega \times {R}_{+}\times {R}\times {R}^d$,
  $$\begin{aligned} \left| g_n(\omega ,t,y,z)-g_n(\omega ,t,y^{'},z^{'})\right| \le n\gamma (t)\left( \left| y-y^{'}\right| +\left| z-z^{'}\right| \right) ; \end{aligned}$$
(iv) *Strong convergence: if* $(y_n,z_n)\rightarrow (y,z)$, *as* $n\rightarrow \infty$, *then*
  $$\begin{aligned} g_n(\omega ,t,y_n,z_n)\rightarrow g(\omega ,t,y,z),\ \ \hbox {as}\ \ n\rightarrow \infty . \end{aligned}$$

The proof of Lemma  6 is very similar to that of Lemma 1 in Lepeltier and San Martin (1997), so we omit it.

We also define the function

$$\begin{aligned} G(\omega ,t,y,z):=\gamma (t)(1+|y|+|z|), \quad \forall (\omega ,t,y,z)\in \Omega \times {R}_{+}\times {R}\times {R}^d. \end{aligned}$$

For each given $\xi \in L^{p}(\Omega ,{\mathcal {F}},P,{R})$, by Theorem  2, there exist two pair of processes $(Y^n,Z^n)$ and (*U*, *V*), which are the solutions to the following BSDEs

25 $$\begin{aligned} Y_t^n= & \xi +\int _t^\infty g_n(s,Y_s^n,Z_s^n)\mathrm{d}s-\int _t^\infty Z_s^n \mathrm{d}W_s, \end{aligned}$$

26 $$\begin{aligned} U_t= & \xi +\int _t^\infty G(s,U_s,V_s)\mathrm{d}s-\int _t^\infty V_s \mathrm{d}W_s, \end{aligned}$$

respectively. From Theorem  4 and Lemma  6, we get

27 $$\begin{aligned} \forall n\ge m,\ \ Y^m\le Y^n\le U,\ \ \hbox {a.s.} \end{aligned}$$

### **Lemma 7**

*There exists a constant* $A>0$ *independent of* *n*, *such that*

$$\begin{aligned}&E\left[ \sup \limits _{t\ge 0}\left| U_t\right| ^p \right] \le A, \quad E\left[ \left( \int _0^\infty \left| V_t\right| ^2\mathrm{d}t\right) ^\frac{p}{2}\right] \le A,\\&E\left[ \sup \limits _{t\ge 0}\left| Y_t^n\right| ^p \right] \le A, \quad E\left[ \left( \int _0^\infty \left| Z_t^n\right| ^2\mathrm{d}t\right) ^\frac{p}{2}\right] \le A, \quad \forall n\in {N}. \end{aligned}$$

### *Proof*

Since (*U*, *V*) is the solution of BSDE (26), there exists a constant $B>0$ independent of *n*, such that

$$\begin{aligned} E\left[ \sup \limits _{t\ge 0}\left| U_t\right| ^p \right] \le B, \quad E\left[ \left( \int _0^\infty \left| V_t\right| ^2\mathrm{d}t\right) ^\frac{p}{2}\right] \le B. \end{aligned}$$

From Inequality (27), we can obtain that for each $n\in {N}$,

$$\begin{aligned} |Y_t^n|^p\le 2^{p-1}\left( |Y_t^1|^p+|U_t|^p\right) . \end{aligned}$$

Thus, there exists a constant $C>0$ independent of *n*, such that

$$\begin{aligned} E\left[ \sup \limits _{t\ge 0}\left| Y_t^n\right| ^p \right] \le C,\ \ \forall n\in {N}. \end{aligned}$$

At last, we prove the boundedness of $E\left[ \left( \int _0^\infty \left| Z_t^n\right| ^2\mathrm{d}t\right) ^\frac{p}{2}\right]$. Applying Itô’s formula to $\left| Y_t^n\right| ^2$, we have

28 $$\begin{aligned}&\left| Y_0^n\right| ^2+\int _0^\infty \left| Z_t^n\right| ^2\mathrm{d}t\nonumber \\&\quad =|\xi |^2+2\int _0^\infty Y_t^n g_n\left( t,Y_t^n,Z_t^n\right) \mathrm{d}t-2\int _0^\infty Y_t^nZ_t^n\mathrm{d}W_t. \end{aligned}$$

By Lemma  6 (i), we know $|g_n(t,y,z)|\le \gamma (t)(1+|y|+|z|)$. Thus, we have

29 $$\begin{aligned} 2\left| Y_t^n g_n\left( t,Y_t^n,Z_t^n\right) \right|\le &\; 2 \gamma (t)\left( \left| Y_t^n\right| +\left| Y_t^n\right| ^2+\left| Y_t^nZ_t^n\right| \right) \nonumber \\\le &\; \gamma (t)\left( 1+\left| Y_t^n\right| ^2\right) +2\gamma (t)\left| Y_t^n\right| ^2\nonumber \\&+2\gamma ^2(t)\left| Y_t^n\right| ^2+\frac{1}{2}\left| Z_t^n\right| ^2\nonumber \\\le &\;\gamma (t)+3\left( \gamma (t)+\gamma ^2(t)\right) \sup \limits _{t\ge 0} \left| Y_t^n\right| ^2+\frac{1}{2}\left| Z_t^n\right| ^2 \end{aligned}$$

It follows that

30 $$\begin{aligned} \int _0^\infty \left| Z_t^n\right| ^2\mathrm{d}t\le\, & 2|\xi |^2+2\int _0^\infty \gamma (t)\mathrm{d}t\nonumber \\&\quad +6\left( \int _0^\infty \gamma (t)\mathrm{d}t+\int _0^\infty \gamma ^2(t)\mathrm{d}t\right) \sup \limits _{t\ge 0} \left| Y_t^n\right| ^2+4\left| \int _0^\infty Y_t^nZ_t^n\mathrm{d}W_t\right| . \end{aligned}$$

Using the following fact: if *b*, $a_i\ge 0$ and $b\le \sum \nolimits _{i=1}^na_i$, then $b^p\le \sum \nolimits _{i=1}^na_i^p$ for any $p\in (0,1)$, we have

31 $$\begin{aligned} \left( \int _0^\infty \left| Z_t^n\right| ^2\mathrm{d}t\right) ^{\frac{p}{2}}\le &\,c_p\left( |\xi |^p+\left( \int _0^\infty \gamma (t)\mathrm{d}t\right) ^{\frac{p}{2}}\right) \nonumber \\&\quad + c_p\left( \int _0^\infty \gamma (t)\mathrm{d}t+\int _0^\infty \gamma ^2(t)\mathrm{d}t\right) ^{\frac{p}{2}}\sup \limits _{t\ge 0} \left| Y_t^n\right| ^p+ c_p\left| \int _0^\infty Y_t^nZ_t^n\mathrm{d}W_t\right| ^{\frac{p}{2}}, \end{aligned}$$

where $c_p$ is a positive constant depending only on *p*. By the Burkholder–Davis–Gundy inequality, we get

32 $$\begin{aligned} c_pE\left[ \left| \int _0^\infty Y_t^nZ_t^n\mathrm{d}W_t\right| ^{\frac{p}{2}}\right]\le &\;d_pE\left[ \left( \int _0^\infty \left| Y_t^nZ_t^n\right| ^2\mathrm{d}t\right) ^\frac{p}{4}\right] \nonumber \\\le &\;d_pE\left[ \sup \limits _{t\ge 0}\left| Y_t^n\right| ^{\frac{p}{2}}\left( \int _0^\infty \left| Z_t^n\right| ^2\mathrm{d}s\right) ^\frac{p}{4}\right] \end{aligned}$$

and thus

33 $$\begin{aligned} c_pE\left[ \left| \int _0^\infty Y_t^nZ_t^n\mathrm{d}W_t\right| ^{\frac{p}{2}}\right]&\le \frac{1}{2}E\left[ \left( \int _0^\infty \left| Z_t^n\right| ^2\mathrm{d}t\right) ^\frac{p}{2}\right] \nonumber \\ &\quad+\frac{d_p^2}{2}E\left[ \sup \limits _{t\ge 0}\left| Y_t^n\right| ^p\right] , \end{aligned}$$

where $d_p$ is a positive constant depending only on *p*. From (31) and (33), we have

34 $$\begin{aligned}&E\left[ \left( \int _0^\infty \left| Z_t^n\right| ^2\mathrm{d}t\right) ^{\frac{p}{2}}\right] \nonumber \\&\quad \le C_p\left[ E[|\xi |^p]+\left( \int _0^\infty \gamma (t)\mathrm{d}t\right) ^{\frac{p}{2}}\right] \nonumber \\&\qquad + C_p\left[ 1+\left( \int _0^\infty \gamma (t)\mathrm{d}t+\int _0^\infty \gamma ^2(t)\mathrm{d}t\right) ^{\frac{p}{2}}\right] E\left[ \sup \limits _{t\ge 0}\left| Y_t^n\right| ^p\right] , \end{aligned}$$

where $C_p$ is a positive constant depending only on *p*. Thus, there exists a constant $A>0$ independent of *n*, such that

$$\begin{aligned}&E\left[ \sup \limits _{t\ge 0}\left| U_t\right| ^p \right] \le A, \quad E\left[ \left( \int _0^\infty \left| V_t\right| ^2\mathrm{d}t\right) ^\frac{p}{2}\right] \le A,\\&E\left[ \sup \limits _{t\ge 0}\left| Y_t^n\right| ^p \right] \le A,\quad E\left[ \left( \int _0^\infty \left| Z_t^n\right| ^2\mathrm{d}t\right) ^\frac{p}{2}\right] \le A,\ \ \forall n\in {N}. \end{aligned}$$

The proof of Lemma  7 is complete. $\square$

### **Lemma 8**

$$\left\{ \left( Y^n,Z^n\right) \right\} _{n=1}^\infty$$ *converges in* $${\mathcal { S}}^p({R})\times {\mathcal { L}}^p({R}^d)$$.

### Proof

Since $\left\{ Y^n\right\} _{n=1}^\infty$ is increasing and bounded in ${\mathcal { S}}^p({R})$, we deduce from the dominated convergence theorem that $Y^n$ converges in ${\mathcal { S}}^p({R})$. We shall denote by *Y* the limit of $Y^n$. Applying Itô’s formula to $\left| Y_t^n-Y_t^m\right| ^2$, we get for any *n*, $m\in {N}$,

35 $$\begin{aligned}&\left| Y_0^n-Y_0^m\right| ^2+\int _0^\infty \left| Z_t^n-Z_t^m\right| ^2\mathrm{d}t\nonumber \\&\quad =2\int _0^\infty \left( Y_t^n-Y_t^m\right) \left( g_n\left( t,Y_t^n,Z_t^n\right) -g_m\left( t,Y_t^m,Z_t^m\right) \right) \mathrm{d}t\nonumber \\&\qquad -2\int _0^\infty \left( Y_t^n-Y_t^m\right) \left( Z_t^n-Z_t^m\right) \mathrm{d}W_t. \end{aligned}$$

Thus, we have

36 $$\begin{aligned}&\int _0^\infty \left| Z_t^n-Z_t^m\right| ^2\mathrm{d}t\nonumber \\&\quad \le 2\sup \limits _{t\ge 0}\left| Y_t^n-Y_t^m\right| \int _0^\infty \left| g_n\left( t,Y_t^n,Z_t^n\right) \right| \mathrm{d}t+2\sup \limits _{t\ge 0}\left| Y_t^n-Y_t^m\right| \int _0^\infty \left| g_m\left( t,Y_t^m,Z_t^m\right) \right| \mathrm{d}t\nonumber \\&\qquad +2\left| \int _0^\infty \left( Y_t^n-Y_t^m\right) \left( Z_t^n-Z_t^m\right) \mathrm{d}W_t\right| . \end{aligned}$$

Using the following fact: if *b*, $a_i\ge 0$ and $b\le \sum \nolimits _{i=1}^na_i$, then $b^p\le \sum \nolimits _{i=1}^na_i^p$ for any $p\in (0,1)$, it follows that

37 $$\begin{aligned}&E\left[ \left( \int _0^\infty \left| Z_t^n-Z_t^m\right| ^2\mathrm{d}t\right) ^{\frac{p}{2}}\right] \nonumber \\&\quad \le c_pE\left[ \sup \limits _{t\ge 0}\left| Y_t^n-Y_t^m\right| ^{\frac{p}{2}}\left( \int _0^\infty \left| g_n\left( t,Y_t^n,Z_t^n\right) \right| \mathrm{d}t\right) ^{\frac{p}{2}}\right] \nonumber \\&\qquad +c_pE\left[ \sup \limits _{t\ge 0}\left| Y_t^n-Y_t^m\right| ^{\frac{p}{2}}\left( \int _0^\infty \left| g_m\left( t,Y_t^m,Z_t^m\right) \right| \mathrm{d}t\right) ^{\frac{p}{2}}\right] \nonumber \\&\qquad +c_pE\left[ \left| \int _0^\infty \left( Y_t^n-Y_t^m\right) \left( Z_t^n-Z_t^m\right) \mathrm{d}W_t\right| ^{\frac{p}{2}}\right] , \end{aligned}$$

where $c_p$ is a positive constant depending only on *p*. From Schwarz’s inequality, we have

38 $$\begin{aligned}&E\left[ \sup \limits _{t\ge 0}\left| Y_t^n-Y_t^m\right| ^{\frac{p}{2}}\left( \int _0^\infty \left| g_k\left( t,Y_t^k,Z_t^k\right) \right| \mathrm{d}t\right) ^{\frac{p}{2}}\right] \nonumber \\\le &\; \left( E\left[ \sup \limits _{t\ge 0}\left| Y_t^n-Y_t^m\right| ^p\right] \right) ^\frac{1}{2} \left( E\left[ \left( \int _0^\infty \left| g_k\left( t,Y_t^k,Z_t^k\right) \right| \mathrm{d}t\right) ^p\right] \right) ^\frac{1}{2},\ \ k=n,m. \end{aligned}$$

By Lemma  6 (i), we can obtain

39 $$\begin{aligned}&E\left[ \left( \int _0^\infty \left| g_k\left( t,Y_t^k,Z_t^k\right) \right| \mathrm{d}t\right) ^p\right] \nonumber \\&\quad \le E\left[ \left( \int _0^\infty \gamma (t)\left( 1+\left| Y_t^k\right| +\left| Z_t^k\right| \right) \mathrm{d}t\right) ^p\right] \nonumber \\&\quad \le d_p\left( \int _0^\infty \gamma (t) \mathrm{d}t\right) ^p+d_p\left( \int _0^\infty \gamma (t) \mathrm{d}t\right) ^pE\left[ \sup \limits _{t\ge 0}\left| Y_t^k\right| ^p\right] \nonumber \\&\qquad +d_p\left( \int _0^\infty \gamma ^2(t) \mathrm{d}t\right) ^\frac{p}{2}E\left[ \left( \int _0^\infty \left| Z_t^k\right| ^2 \mathrm{d}t\right) ^\frac{p}{2}\right] ,\ \ k=n,m \end{aligned}$$

where $d_p$ is a positive constant depending only on *p*. Thus, by Lemma  7, there exists a constant $D>0$ independent of *n*, *m* such that

40 $$\begin{aligned}&E\left[ \sup \limits _{t\ge 0}\left| Y_t^n-Y_t^m\right| ^{\frac{p}{2}}\left( \int _0^\infty \left| g_n\left( t,Y_t^n,Z_t^n\right) \right| \mathrm{d}t\right) ^{\frac{p}{2}}\right] \le D\left( E\left[ \sup \limits _{t\ge 0} \left| Y_t^n-Y_t^m\right| ^p\right] \right) ^\frac{1}{2}, \end{aligned}$$

41 $$\begin{aligned}&E\left[ \sup \limits _{t\ge 0}\left| Y_t^n-Y_t^m\right| ^{\frac{p}{2}}\left( \int _0^\infty \left| g_m\left( t,Y_t^m,Z_t^m\right) \right| \mathrm{d}t\right) ^{\frac{p}{2}}\right] \le D\left( E \left[ \sup \limits _{t\ge 0}\left| Y_t^n-Y_t^m\right| ^p\right] \right) ^\frac{1}{2}. \end{aligned}$$

By the Burkholder–Davis–Gundy inequality, we get

42 $$\begin{aligned}&c_pE\left[ \left| \int _0^\infty \left( Y_t^n-Y_t^m\right) \left( Z_t^n-Z_t^m\right) \mathrm{d}W_t\right| ^{\frac{p}{2}}\right] \nonumber \\&\quad \le e_pE\left[ \left( \int _0^\infty \left| Y_t^n-Y_t^m\right| ^2\left| Z_t^n-Z_t^m\right| ^2\mathrm{d}t\right) ^\frac{p}{4}\right] \nonumber \\&\quad \le e_pE\left[ \sup \limits _{t\ge 0}\left| Y_t^n-Y_t^m\right| ^{\frac{p}{2}}\left( \int _0^\infty \left| Z_t^n-Z_t^m\right| ^2\mathrm{d}t\right) ^\frac{p}{4}\right] \end{aligned}$$

and thus

43 $$\begin{aligned} c_pE\left[ \left| \int _0^\infty \left( Y_t^n-Y_t^m\right) \left( Z_t^n-Z_t^m\right) \mathrm{d}W_t\right| ^{\frac{p}{2}}\right]\le &\; \frac{1}{2}E\left[ \left( \int _0^\infty \left| Z_t^n-Z_t^m\right| ^2\mathrm{d}t\right) ^\frac{p}{2}\right] \nonumber \\&\quad +\frac{e_p^2}{2}E\left[ \sup \limits _{t\ge 0}\left| Y_t^n-Y_t^m\right| ^p\right] , \end{aligned}$$

where $e_p$ is a positive constant depending only on *p*. From (37), (40), (41) and (43), we have

44 $$\begin{aligned}&E\left[ \left( \int _0^\infty \left| Z_t^n-Z_t^m\right| ^2\mathrm{d}t\right) ^{\frac{p}{2}}\right] \nonumber \\&\quad \le C_p\left( \left( E\left[ \sup \limits _{t\ge 0}\left| Y_t^n-Y_t^m\right| ^p\right] \right) ^\frac{1}{2}+ E\left[ \sup \limits _{t\ge 0}\left| Y_t^n-Y_t^m\right| ^p\right] \right) , \end{aligned}$$

where $C_p$ is a positive constant depending only on *p*. Thus, $\left\{ Z^n\right\} _{n=1}^\infty$ is a Cauchy sequence in $\mathcal{L}^p({R}^d)$, from which the result follows. The proof of Lemma  8 is complete.

### Proof of Theorem  5

For all $n\in {N}$, we have $Y^n\le U$, and $\left\{ Y^n\right\} _{n=1}^\infty$ converges in ${\mathcal { S}}^p({R})$, $\mathrm{d}t\times \mathrm{d}P$-a.s. to $Y\in {\mathcal { S}}^p({R})$.

On the other hand, since $Z^n$ converges in ${\mathcal { L}}^p({R}^d)$ to *Z*, we can assume, choosing a subsequence if needed, that $Z^n\rightarrow Z$, $\mathrm{d}t\times \mathrm{d}P$-a.s., as $n\rightarrow \infty$ and $\overline{G}:=\sup \nolimits _{n}|Z^n|$ is $\mathrm{d}t\times \mathrm{d}P$ integrable. Therefore, by Lemma  6 (i) and (iv), we get for almost all $\omega$,

45 $$\begin{aligned}&g_n\left( t,Y_t^n,Z_t^n\right) \rightarrow g(t,Y_t,Z_t),\ \ \mathrm{d}t-\hbox {a.e.},\ \ \hbox {as}\ \ n\rightarrow \infty , \nonumber \\&\left| g_n\left( t,Y_t^n,Z_t^n\right) \right| \le \gamma (t)\left( 1+\left| Y_t^n\right| +\left| Z_t^n\right| \right) \nonumber \\&\quad\quad \quad\quad\quad\quad\le \gamma (t)\left( 1+\sup \limits _{n}\left| Y_t^n\right| +\overline{G}_t\right) \in L^1([0,\infty );\mathrm{d}t). \end{aligned}$$

Thus, for almost all $\omega$ and uniformly in *t*, it holds that

$$\begin{aligned} \int _t^\infty g_n\left( s,Y_s^n,Z_s^n\right) \mathrm{d}s\rightarrow \int _t^\infty g(s,Y_s,Z_s)\mathrm{d}s,\quad \hbox {as}\ \ n\rightarrow \infty . \end{aligned}$$

From the continuity properties of the stochastic integral, it follows that

$$\begin{aligned} \sup \limits _{t\ge 0} \left| \int _t^\infty Z_s^n\mathrm{d}W_s-\int _t^\infty Z_s\mathrm{d}W_s\right| \rightarrow 0\ \ \hbox {in probability},\ \ \hbox {as}\ \ n\rightarrow \infty . \end{aligned}$$

Choosing again, a subsequence, we can assume that the above convergence is *P*-a.s. Finally,

46 $$\begin{aligned} \left| Y_t^n-Y_t^m\right|\le & \int _t^\infty \left| g_n\left( s,Y_s^n,Z_s^n\right) -g_m\left( s,Y_s^m,Z_s^m\right) \right| \mathrm{d}s\nonumber \\&\quad +\left| \int _t^\infty \left( Z_s^n-Z_s^m\right) \mathrm{d}W_s\right| , \end{aligned}$$

and taking limits on *m* and supremum over *t*, we get

47 $$\begin{aligned} \sup \limits _{t\ge 0}\left| Y_t^n-Y_t\right|\le & \int _0^\infty \left| g_n\left( s,Y_s^n,Z_s^n\right) -g\left( s,Y_s,Z_s\right) \right| \mathrm{d}s\nonumber \\&\quad +\sup \limits _{t\ge 0}\left| \int _t^\infty \left( Z_s^n-Z_s\right) \mathrm{d}W_s\right| ,\ \ P-\hbox {a.s.} \end{aligned}$$

from which it follows that $Y^n$ converges uniformly in *t* to *Y* (in particular, *Y* is a continuous process). Note that $\left\{ Y^n\right\} _{n=1}^\infty$ is monotone; therefore, we actually have the uniform convergence for the entire sequence and not just for a subsequence. Taking limits in Equation (25), we deduce that (*Y*, *Z*) is a solution of BSDE (2).

Let $(\tilde{Y},\tilde{Z})\in {\mathcal { S}}^p({R})\times {\mathcal { L}}^p({R}^d)$ be any solution of BSDE (2). From Theorem  4, we get that $Y^n\le \tilde{Y}$, $\forall n\in {N}$ and therefore $Y\le \tilde{Y}$ proving that *Y* is the minimal solution. The proof of Theorem  5 is complete. $\square$

### Remark 9

By Theorem  5, we have: Under the assumption (A.3) and (A.4), for each given $\xi \in {\mathcal { L}}(\Omega ,{\mathcal {F}},P,{R})$, BSDE (2) has a solution $(Y,Z)\in {\mathcal { S}}({R})\times {\mathcal { L}}({R}^d)$. Also, in ${\mathcal { S}}({R})\times {\mathcal { L}}({R}^d)$, there is a minimal solution $(\underline{Y},\underline{Z})$ of BSDE (2), in the sense that for any other solution (*Y*, *Z*) of (2), we have $\underline{Y}\le Y$.

## Conclusion

In this paper, we have solved two problems on infinite time interval BSDEs. Firstly, by using an a priori estimate (Lemma  3), we studied the existence and uniqueness theorem for $L^{p}$ $(1<p<2)$ solutions of infinite time interval BSDEs with non-uniformly Lipschitz coefficients (Theorem  2). It should be pointed out that the assumptions of Theorem  2 is weaker than that of Theorem 3.1 in Zong (2013). Secondly, applying comparison theorem for 1-dimensional infinite time interval BSDEs (Theorem  4), we studied the existence theorem for $L^{p}$ $(1<p<2)$ solutions of 1-dimensional infinite time interval BSDEs under the conditions that the coefficients are continuous and have linear growths (Theorem  5). In Theorem  5, the existence of a minimal solution was also obtained.
